# Combining Public Health Education and Disease Ecology Research: Using Citizen Science to Assess Chagas Disease Entomological Risk in Texas

**DOI:** 10.1371/journal.pntd.0004235

**Published:** 2015-12-10

**Authors:** Rachel Curtis-Robles, Edward J. Wozniak, Lisa D. Auckland, Gabriel L. Hamer, Sarah A. Hamer

**Affiliations:** 1 Department of Veterinary Integrative Biosciences, Texas A&M University, College Station, Texas, United States of America; 2 Texas Department of State Health Services, Uvalde, Texas, United States of America; 3 Texas State Guard Medical Brigade, Uvalde, Texas, United States of America; 4 Department of Entomology, Texas A&M University, College Station, Texas, United States of America; Universidad de Buenos Aires, ARGENTINA

## Abstract

**Background:**

Chagas disease is a zoonotic parasitic disease well-documented throughout the Americas and transmitted primarily by triatomine ‘kissing bug’ vectors. In acknowledgment of the successful history of vector control programs based on community participation across Latin America, we used a citizen science approach to gain novel insight into the geographic distribution, seasonal activity, and *Trypanosoma cruzi* infection prevalence of kissing bugs in Texas while empowering the public with information about Chagas disease.

**Methodology/Principal Findings:**

We accepted submissions of kissing bugs encountered by the public in Texas and other states from 2013–2014 while providing educational literature about Chagas disease. In the laboratory, kissing bugs were identified to species, dissected, and tested for *T*. *cruzi* infection. A total of 1,980 triatomines were submitted to the program comprised of at least seven species, of which *T*. *gerstaeckeri* and *T*. *sanguisuga* were the most abundant (85.7% of submissions). Triatomines were most commonly collected from dog kennels and outdoor patios; Overall, 10.5% of triatomines were collected from inside the home. Triatomines were submitted from across Texas, including many counties which were not previously known to harbor kissing bugs. Kissing bugs were captured primarily throughout April-October, and peak activity occurred in June-July. Emails to our dedicated account regarding kissing bugs were more frequent in the summer months (June-August) than the rest of the year. We detected *T*. *cruzi* in 63.3% of tested bugs.

**Conclusions/Significance:**

Citizen science is an efficient approach for generating data on the distribution, phenology, and infection prevalence of kissing bugs—vectors of the Chagas disease parasite—while educating the public and medical community.

## Author Summary

We created a kissing bug citizen science program in Texas to educate the public about Chagas disease, a vector-borne disease of humans and dogs, and to create a mechanism for the public to submit triatomine ‘kissing bug’ vectors to our research program. From May 2013 to December 2014, we designed an interactive website, distributed pamphlets, and responded to emails and phone calls from the public. This resulted in the submission of 1,980 kissing bugs, mostly collected from dog kennels and outdoor patios, expanding the geographic regions known to harbor kissing bugs in Texas and allowing insight into a cross-section of bugs of high epidemiological and veterinary relevance. Citizen submissions of kissing bugs peaked in June-July and showed 63.3% infection prevalence with *Trypanosoma cruzi*. Citizen science is an efficient mechanism for gaining novel insight into vector occurrence and infection.

## Introduction

Citizen science—the engagement of non-scientists in collecting scientific data—has long been used in ecological and wildlife research [1–3], resulting in an engaged public and providing researchers access to data from large geographic and temporal scales. We pose that citizen science is a powerful yet underutilized tool in public health, given that community engagement is recognized as a core component of many successful public health programs [4].

**Fig 1:**
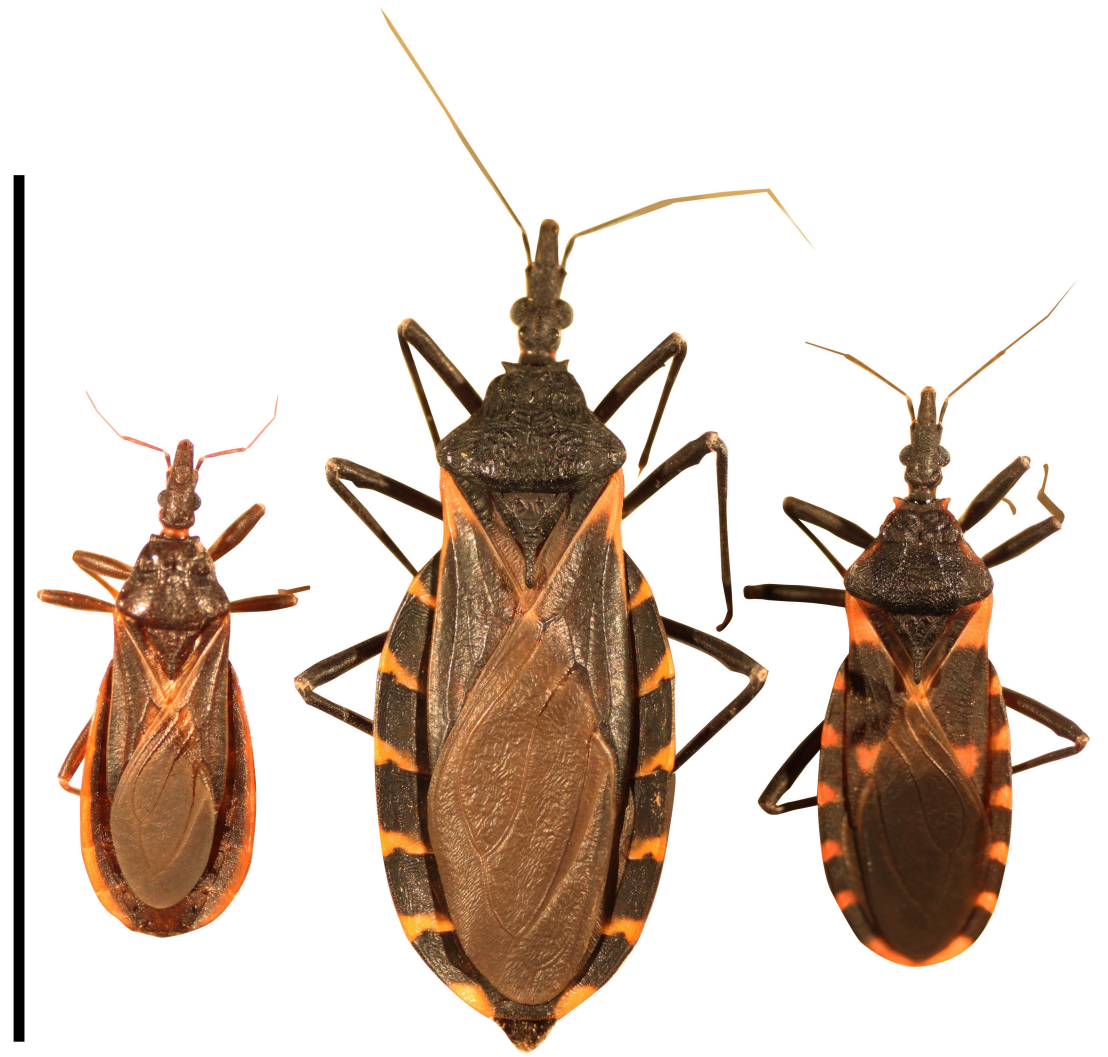
Three species of kissing bugs commonly found in Texas. (Left to right) *Triatoma protracta*, the most common species in the western U.S.; *Triatoma gerstaeckeri*, the most common species in Texas; *Triatoma sanguisuga*, the most common species in the eastern U.S. Scale bar represents 25mm or approximately 1 inch.

Chagas disease is a vector-borne zoonotic disease caused by the parasitic protozoan *Trypanosoma cruzi*. Infection with *T*. *cruzi* can result in cardiac and digestive disease in humans and dogs that may not manifest until years after infection. Disease in humans is well-documented throughout the Americas [5,6], and canine Chagas disease is well-documented in Texas [7,8]. In 2013 and 2014, the first two years in which Chagas disease was a notifiable disease in Texas, a total of 351 canine cases and 39 human cases were reported; the latter including 12 locally-acquired cases [9,10]. Colloquially referred to as ‘kissing bugs’, triatomine insects (Fig 1) are vectors of *Trypanosoma cruzi*. In the U.S., the highest species diversity of triatomines is found in Texas [5,11]. Triatomine bugs feed on blood during all stages of their lives, and may acquire the parasite from feeding on an infected mammal. The parasite *T*. *cruzi* is spread through the feces of the insect.

Community-based vector surveillance has been widespread for decades as an approach to manage Chagas disease in South and Central America, through which householders monitor kissing bug presence within the home to allow for timely response with insecticide treatment. In these regions, some species of kissing bugs occupy a domestic niche (i.e., they successfully establish colonies in houses) [12]. Diverse approaches have been employed in community-based vector surveillance programs, including the use of sensor boxes for passive detection of triatomines [13] and training of community leaders in monitoring for reinfestation and insecticide spraying [14,15]. Community-based collections were found to be more sensitive than the gold standard of timed manual searches for triatomine recoveries [16]. A systematic review of Chagas disease vector control interventions across South and Central America concluded that community participatory surveillance significantly boosted vector detection probabilities above those found by vector control program staff using active searches or vector detection devices [17]. Further, retrospective analyses of data from Argentina revealed that vector control strategies that incorporate community participation avert more human cases of disease and cost less than vertical or centralized strategies that consist of insecticide application by program staff only [18].

Community engagement has less commonly been used in the southern U.S. for kissing bug research and control, likely because the vector species in the southern U.S. tend not to colonize homes in the same manner as in Latin America, and insecticide spraying within the home is therefore not a widely used tool for public health protection. The first recruitment of the public in the U.S. to help collect kissing bugs was in 1941, when Dr. Sherwin F. Wood of Los Angeles City College encouraged Arizona miners to collect insects from their sleeping quarters with the recruitment slogan ‘Nab that bug at one cent each for Dr. Wood at City College to keep.’ [19]; this was followed by other similar efforts in the 1940s [20,21]. Subsequently, community participation in kissing bug surveillance in Tucson, Arizona [22,23] and New Orleans, Louisiana [24] has provided unprecedented information on vector phenology and infection in these regions. For public health and research purposes, the recruitment of submissions of kissing bugs from citizens who incidentally encounter them is an attractive option for collection given that kissing bugs are nocturnal, elusive, and difficult to collect using standardized entomological traps [11,25,26]. Akin to the widespread community-based vector surveillance programs in areas where Chagas disease is endemic across Latin America, here, we describe the implementation and results from a two-year citizen science program in Texas that provides public health education and encourages citizens to aid in the collection of kissing bugs across Texas.

## Materials and Methods

In early 2013, we developed a citizen science program for Chagas disease research with priority interest in Texas. Our program provides resources for people seeking information about Chagas disease and kissing bugs in the U.S., while also requesting kissing bug samples through a variety of media: printed pamphlets (S1 File and S2 File), phone communication, an educational website (http://kissingbug.tamu.edu), solicitations on news stations, and a dedicated e-mail address (kissingbug@cvm.tamu.edu). The public may submit insect photos through the website or email to be identified by our team, and are invited to submit kissing bug specimens along with associated information. Submitters are informed about Chagas disease transmission, and cautioned to not touch the insects with bare hands. We request that the bugs be captured in bags and stored in a freezer, to kill the insect before shipping. The minimum requested dataset to accompany each bug includes: date, time and location of capture and whether the bug was alive or dead. Location data were validated and geo-coded in a geographic information system (ArcMap, ESRI, Redlands, CA).

In the laboratory, kissing bugs were identified to species [27], measured, sexed, and dissected. Following DNA extraction (Omega Bio-tek, Norcross, GA; Qiagen, Germantown, MD), bug hindguts were tested for infection through amplification of *T*. *cruzi* satellite DNA quantitative real-time PCR that is selective for *T*. *cruzi* [28] for which our internal validations defined a positive sample as one with a cycle threshold value of 33 or less. This qPCR is highly sensitive with a limit of detection that approximates 0.5 parasite equivalents of DNA [28]. Bugs known to have fed on humans were sent to the Centers for Disease Control via Texas Department of State Health Services for testing so that submitters can be in immediate contact with those who can make medical recommendations. We provided submitters with the species identification and preliminary *T*. *cruzi* infection status of their submission, which is shared with a statement about potential false positive or false negative results. Our website includes an interactive map to allow submitters to see their data contributions and examine the spatial and temporal distribution of kissing bugs submitted by the public Texas. We provided citizens with information on reducing kissing bug occurrence in dog kennels or patios outside the home, as these were the primary areas from which bugs were collected. These recommendations included turning off the outdoor lights at night, housing dogs indoors when possible, reducing woody debris or other potential bug harborage sites within the vicinity of the home/kennel, and the use of commercially-available insecticides, although none available in the United States are labeled for the control of triatomines.

## Results

From May 2013 through December 2014, we received approximately 4,000 emails that resulted in a total of 1,980 kissing bug submissions. The triatomines submitted to the program comprised at least seven species, of which *T*. *gerstaeckeri* and *T*. *sanguisuga* were most abundant (71.3 and 14.4% of submissions, respectively; Fig 1 and Table 1). Locations from which triatomines were most commonly reported to be collected include dog kennels (24.6%), patios/porches (19%), and inside homes (10.8%), followed by other locations including outside walls of homes, garages, cat sleeping areas, inside buildings, barns, pools, tents, and chicken coops. Overall, 10.8% of triatomines representing 5 species were collected from inside homes (Table 1) and the majority of submissions of adults from inside homes consisted of a single bug that was encountered. As a proportion of the number of collections of each species, *T*. *rubida*, *T*. *lecticularia*, and *T*. *sanguisuga* were most commonly captured inside homes. Regarding nymphs, 20 of the 56 (36.7%) nymphs submitted to our program were collected from inside the home (Table 1), including three submissions of more than one nymph (two submissions with two nymphs; one submission with five nymphs).

**Table 1:** Triatomine species, proportion encountered inside homes, and *T*. *cruzi* infection prevalence in bugs submitted to the Texas Citizen Science kissing bug program, 2013–2014.

			*T*. *cruzi* infection prevalence	
Species	No. submitted (% of total)	No. from inside house (% of species)	No. tested	No. positive (% of species)
Adults				
*T*. *gerstaeckeri*	1412 (71.3)	95 (6.7)	487	330 (67.8)
*T*. *indictiva*	108 (5.5)	15 (13.9)	40	17 (42.5)
*T*. *lecticularia*	49 (2.5)	12 (24.5)	20	15 (75)
*T*. *neotomae*	1 (0.1)	0	0	
*T*. *protracta*	2 (0.1)	0	0	
*T*. *rubida*	13 (0.7)	6 (46.2)	6	1 (16.7)
*T*. *sanguisuga*	286 (14.4)	65 (22.7)	120	70 (58.3)
Unknown spp.^a^	53 (2.7)	1 (1.9)	6	3 (50)
Nymphs				
Unknown spp.^b^	56 (2.8)	20 (35.7)	15	3 (20)
Total	1980	214 (10.8)	694	439 (63.3)

^a^Specimens could not be identified to species due to poor quality (smashed bug; missing key morphologic features).

^b^Nymphs were not identified to species.

**Fig 2:**
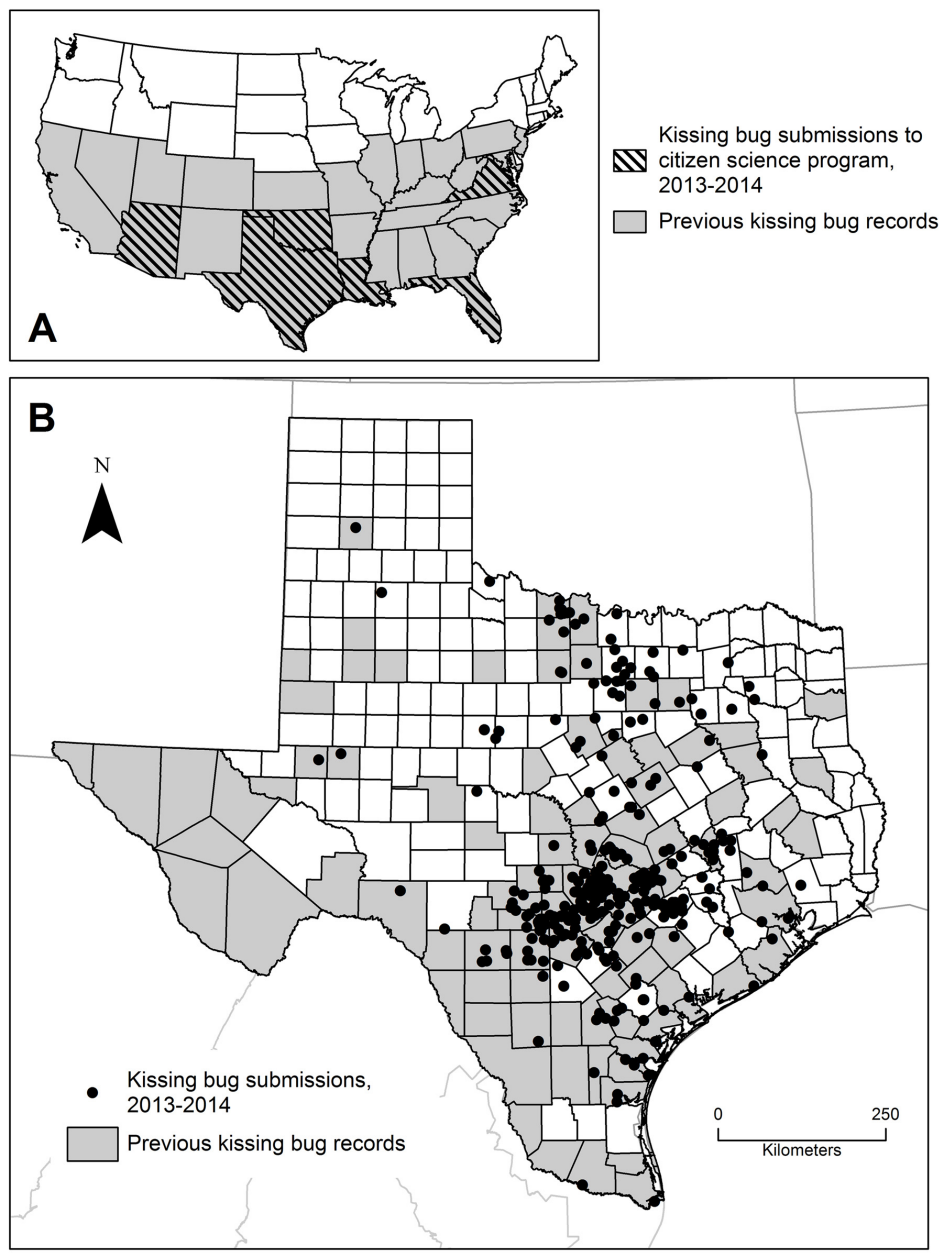
Historical and current collections from across Texas. A) States from which our program received kissing bugs in 2013–2014 overlaid on historical state-level records of kissing bugs throughout the U.S. [5,29]; B) Historical county-level records of kissing bugs in Texas (1928–2006, as from [11]) and submissions of kissing bugs through our citizen science program (2013–2014).

Over 99% of submissions were from Texas (1,968 kissing bugs), although we also received kissing bugs from Arizona, Florida, Louisiana, Oklahoma, and Virginia (Fig 2). In our initial program year in 2013, we received 881 kissing bugs submitted by 145 citizens. Our expanded program in 2014 resulted in the submission of 1,099 kissing bugs by 243 citizens, 13 of which had submitted bugs to our laboratory the preceding year. Whereas the majority of citizens submitted a single bug (200 individuals), many individuals submitted multiple bugs. There were 21 individuals who submitted 20 or more bugs over two years, including one individual who submitted 271 bugs. The majority of these large quantity submitters (90.5%) found bugs in the sleeping quarters of their dogs and expressed concerns about canine Chagas disease risk.

**Fig 3:**
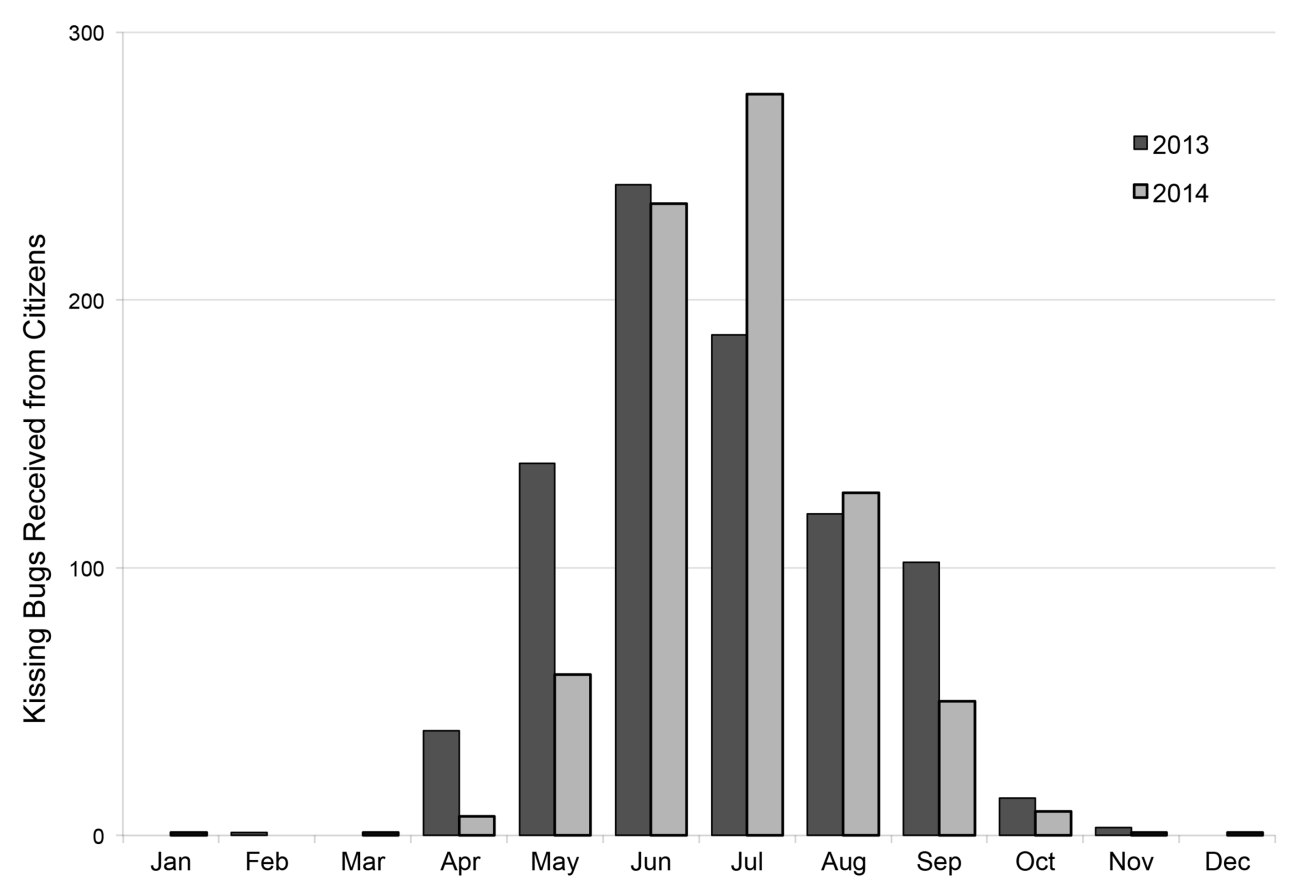
Kissing bug collection phenology. Seasonal occurrence of the collection of kissing bugs by citizens, 2013–2014.

Kissing bugs received by our laboratory were captured primarily throughout April-October, with the highest number of captures in June-July (Fig 3). The small number of kissing bugs (n = 11) that were captured in the winter months (November-March) were mainly collected from indoors (63.6%) and were comprised of a higher percentage of nymphs (36.4%) than submissions throughout the summer months (26.3%). In the subset of 694 kissing bugs submitted through this program that we subjected to molecular detection of *T*. *cruzi*, we detected infection in 493 bugs (63.3%). In all counties from which at least two bugs were submitted, at least one infected bug was detected.

**Fig 4:**
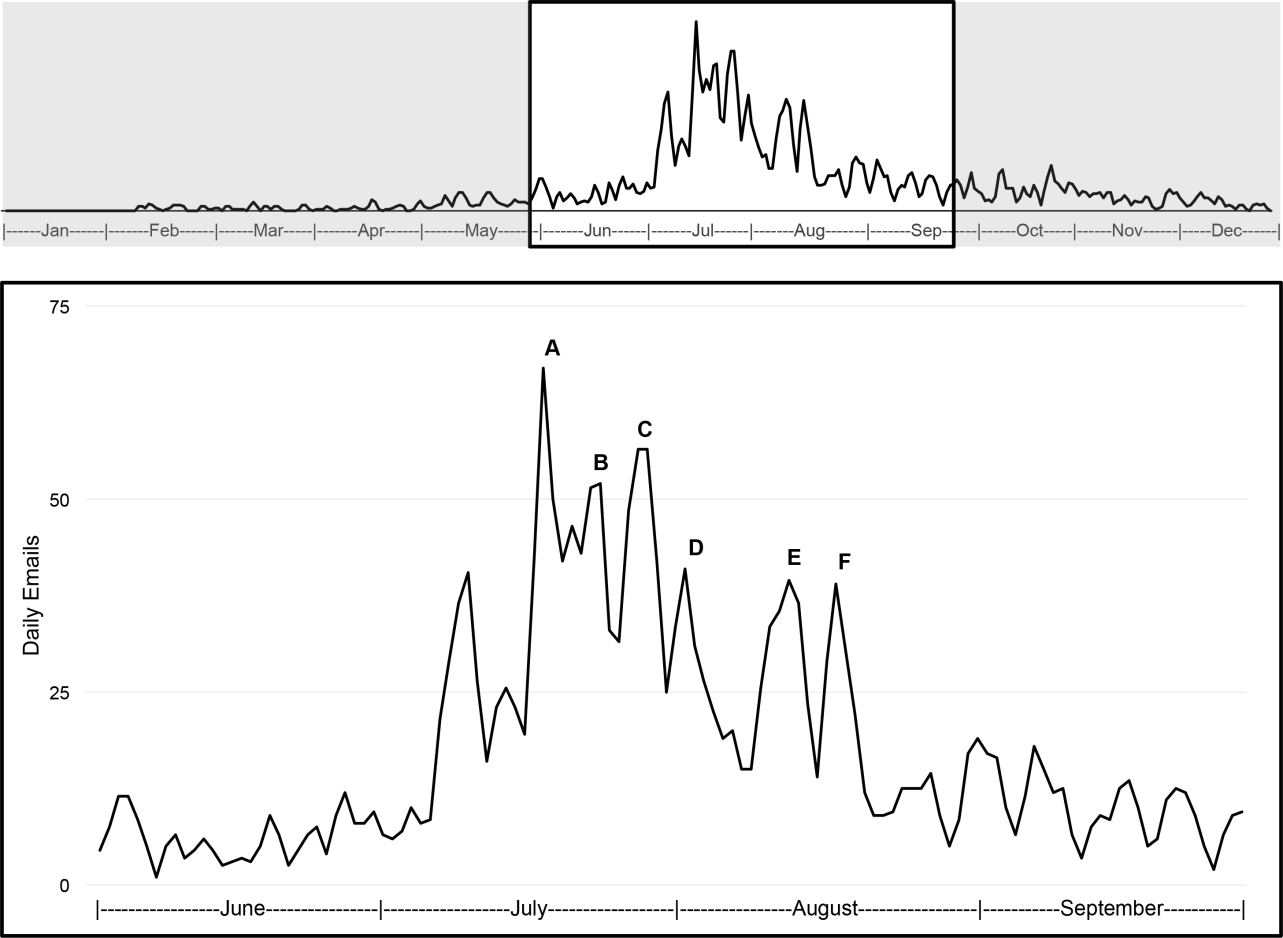
Email activity. Number of daily emails to kissingbug@cvm.tamu.edu from the public, 2014. Peaks generally correspond with a media event featuring Chagas disease and/or kissing bugs: A) July 17/18, Amarillo, Texas newscast and National Public Radio website article; B) July 24, articles about Chagas disease in Virginia; C) July 27, USA Today online article; D) August 2, Arkansas newscast; E) August 11, Cat Channel online article; F) August 18, Lubbock, Texas newscast. The regular pattern of decreasing and increasing activity (most noticeable throughout September) corresponds with weekends and weekdays, respectively.

**Fig 5:**
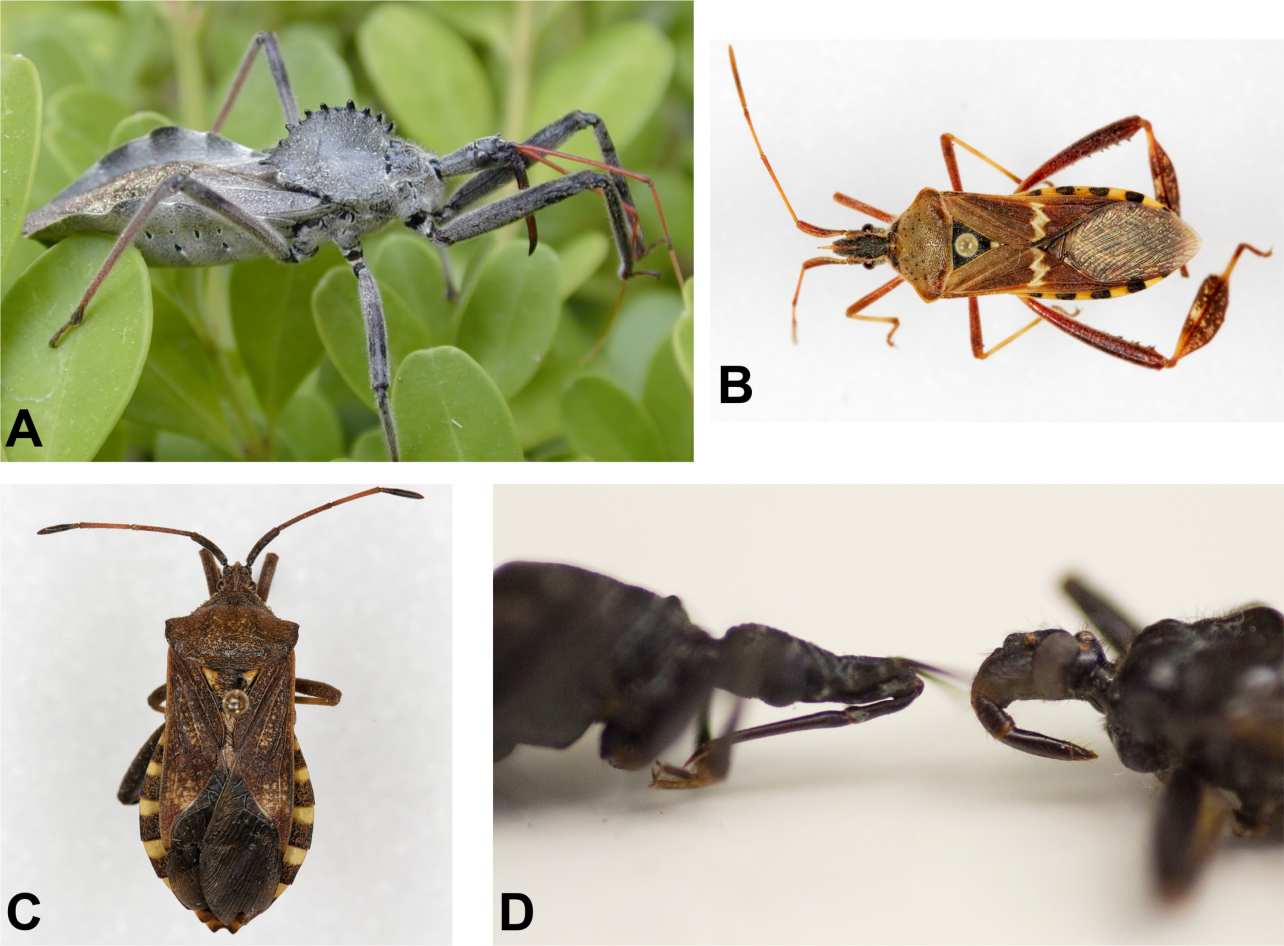
Key features of non-kissing bugs. Key morphologic features distinguish similar-looking insects: (A) Gray color and dorsal crest of wheel bugs (*Arilus cristatus*) (B) Wide, flattened back legs of leaf-footed bugs (*Leptoglossus sp*.) (C) Short head of squash bugs (*Mozena sp*.) (D) Close view of mouthparts of a kissing bug (left; thin and straight) and non-kissing bug (right; thick and curved); Photos courtesy of M. Merchant (A), P. Porter (B, C), and R. Bardin (D).

Since establishing a dedicated email account in late 2013, emails regarding kissing bugs were more frequent in the summer months (June-August) than the rest of the year. Periods of exceptionally high email traffic were frequently associated with newscasts and releases of online media related to Chagas disease and kissing bugs (Fig 4). Captures of non-kissing bugs (mainly reduvvids and other hemipterans; Fig 5) represented approximately 10% of photo submissions to our program. We occasionally received emails from citizens concerned about a bug bite that may be from a triatomine, sometimes accompanied by photos of the bite site. Rarely, these citizens also have collected a kissing bug. In all these cases, we put the citizen in contact with the local contact of the Texas Department of State Health Services who can investigate further and provide medical recommendations when warranted.

## Discussion

We used a citizen science approach to establish a collection of triatomine vectors nearing 2,000 specimens in order to define key periods of kissing bug activity, expand the county-level known range of kissing bugs in Texas, and ascertain infection prevalence with *T*. *cruzi*. This method of sampling provides unique insight into the specific subset of bugs in nature that are epidemiologically relevant—that is, those bugs that people are encountering during daily activities and that potentially pose the highest risk for transmission of *T*. *cruzi* [26]. Further, the educational campaign and community engagement at the core of this initiative allow people to take an active role in understanding how to improve their health.

This two-year citizen science program has revealed a similar geographic distribution of kissing bugs in Texas as was previously documented over almost eight decades (Fig 2). Our results highlight kissing bug occurrence in central and south Texas, which were predicted to be the highest Chagas disease risk zones in a statewide risk map [30] and further extend potential risk zones to include north Texas. Further, the expansive occurrence data from the citizen science initiative can provide unprecedented spatial resolution to complement the limited data used in a previous state-wide effort (108 kissing bugs from 63 unique spatial cells) [30]. We received vector submissions from six of the seven focal areas across Texas from which *T*. *cruzi* seropositive dogs were recently detected [8].

The peak in the collection of adult bugs occurred in June-July, with most activity occurring between April-October. While this apparent phenology certainly reflects periods of human outdoor activity given the collection method, it is congruent with the only other study of triatomine phenology in Texas which employed blacklight traps (a collection method that is independent of public outdoor activity) in a central Texas county in April-September to conclude that 83.4% of adult triatomines collections occurred in May-July [31]. Because vector activity is a key component of human risk, detailed phenology data are useful for public health initiatives.

The detected *T*. *cruzi* infection prevalence in citizen-submitted Texas kissing bugs was 63%, and is similar to that found in previous studies of kissing bugs from Texas that were collected using other means. For example, a sample of 241 bugs, including those collected from wildlife nests and by health department employees, was characterized by 50.7% infection prevalence [11], and 69–82% of bugs collected from houses and dog kennels in central Texas were infected [25].

The analysis of citizen-collected data presents unique challenges due to observer error and sampling bias [3]. For example, public submission programs result non-target bug species [32,33]; however this potential source of observer error is controlled for in our program by laboratory identification of all kissing bugs. The geographic breadth of submissions reflects the area over which citizens are aware of the program and able to contribute to it. While areas from which no bugs were submitted cannot be interpreted as an absence of kissing bugs, the occurrence data are useful for increasing medical and veterinary awareness for Chagas disease over an expanded region.

The longitudinal pattern of inquiries from the public revealed that emails to our citizen science account peaked after media events (Fig 3); many of these emails concerned inaccurate information on television, internet, or social media. The most common cause of confusion resulted when pictures of common bug species that share some similarity in appearance to kissing bugs, but are not vectors of *T*. *cruzi*, were displayed while discussing Chagas disease on the news. Our data demonstrate the influence of the media for increasing awareness for the citizen science initiative to contribute to the growing field of digital epidemiology [34].

This citizen science program has resulted in strengthened relationships among university researchers, state health departments, the Centers for Disease Control and Prevention, clinical veterinarians, medical practitioners, and the general public. Such coordinated efforts among stakeholders—including the public—for insect surveillance offer opportunities for integrated pest management, research, and the protection of human health (e.g., The Collaborative Strategy on Bed Bugs [35] and nuisance black fly reporting [36]). The collection of samples generated through this program will be available for analyses of triatomine population genetics, blood meal analyses, genetic typing of *T*. *cruzi*, and additional research pursuits to enhance our understanding of vector ecology, allowing us to further build upon state-wide and regional models of triatomine distributions and disease risk [30,37]. Given the demonstrated public health benefit of community engagement in vector surveillance and control in areas of Chagas disease endemicity across Latin America, citizen science should be promoted as a key approach for enhancing vector-borne disease research and public health protection efforts in the United States.

## Supporting Information

S1 File Kissing bug pamphlet. Tri-fold pamphlet about kissing bugs and Chagas diesase used in support of citizen science initiative in Texas.
(PDF)

S2 File Canine Chagas disease pamphlet. Tri-fold pamphlet about canine Chagas disease used in support of citizen science initiative in Texas.
(PDF)
