# Supplementary material for: Combining Public Health Education and Disease Ecology Research: Using Citizen Science to Assess Chagas Disease Entomological Risk in Texas
Source: PLoS Negl Trop Dis. 2015 Dec 10;9(12):e0004235. doi: 10.1371/journal.pntd.0004235 (PMC4687635; doi:10.1371/journal.pntd.0004235)
Supplement: S1 File — Tri-fold pamphlet about kissing bugs and Chagas diesase used in support of citizen science initiative in Texas. (PDF) [file pntd.0004235.s001.pdf]

What to do

if you find a kissing bug

Never touch a kissing bug with a bare hand. The parasite they may harbor can be transmitted to humans and other animals. If you see a bug you believe is a kissing bug and would like confirmation of the species identity and to submit it for testing, our lab accepts carefully obtained samples.

Use a glove or small plastic bag to catch the bug to avoid direct contact with the bug. Store the bug in a sealed plastic bag, in a vial, or other small container, all of which are acceptable. All surfaces with which the bug came into contact should be thoroughly cleaned with a bleach solution. You may contact us (information on back) with questions or about submitting a kissing bug.

It is very important for us to know:

- **exactly where the bug was found**
- **the date**
- **the time of day**
- **if the bug was alive when found**
- **what the bug was doing**

Please write down this information to send with the bug.

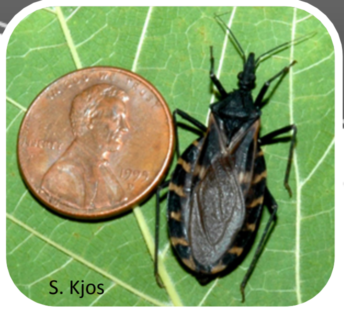

Triatoma gerstaeckeri
Adult female

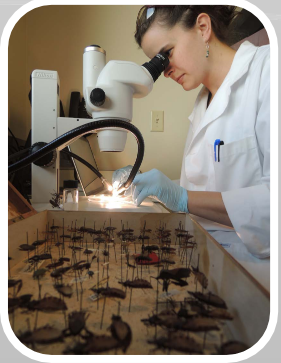

Kissing Bug Research at Texas A&M University

Researchers in the Department of Veterinary Integrative Biosciences and Department of Entomology have new projects underway to understand the ecology of kissing bugs and the parasite that causes Chagas disease. Samples from bugs and potentially infected animals are tested for the parasite, and the results are being used to answer questions related to whether Chagas disease will become a disease of major public health concern in the United States.

Research Team at Texas A&M

http://KissingBug.tamu.edu

KissingBug@cvm.tamu.edu

(979) 458-4924

Graduate Student

- Rachel Curtis, NSF Graduate Research Fellow

Principal Investigators

- Sarah Hamer, PhD, DVM, Assistant Professor
- Gabriel Hamer, PhD, Clinical Assistant Professor
- Karen Snowden, DVM, DACVM, Professor

VETERINARY MEDICINE & BIOMEDICAL SCIENCES
TEXAS A&M UNIVERSITY

AGRICULTURE & LIFE SCIENCES
TEXAS A&M UNIVERSITY

Kissing bugs and Chagas disease

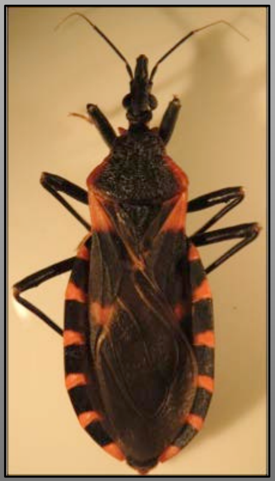
Adult female
Triatoma sanguisuga

VETERINARY MEDICINE & BIOMEDICAL SCIENCES
TEXAS A&M UNIVERSITY

"Discovering tomorrow's health solutions today."

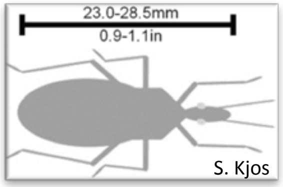

Triatoma gerstaeckeri
Adult male

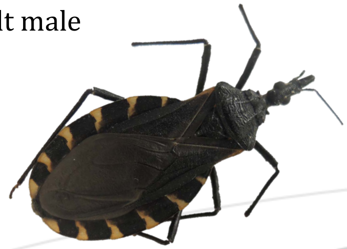

About Kissing Bugs

Kissing bugs are insects also commonly known as cone-nose bugs or *chinches*. Kissing bugs feed on blood during the night, and they are called kissing bugs because they prefer to bite humans around the mouth or eyes. There are 11 different species of kissing bugs in United States. The most common species in the south-central US are *Triatoma sanguisuga* (see picture on front) and *Triatoma gerstaeckeri* (see picture to the left), which are each about 1 inch long. The kissing bugs can be found around woodpiles, debris in the yard, and woodrat nests, but are also attracted to the lights and carbon dioxide that are associated with human houses and dog kennels.

Why worry about Kissing Bugs?

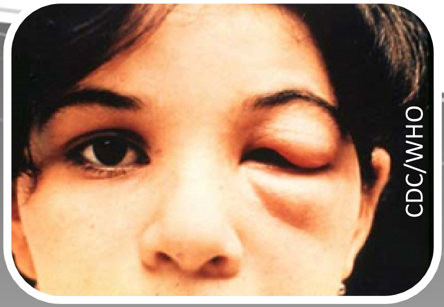

Kissing Bugs & Chagas Disease

Kissing bugs can harbor a parasite called *Trypanosoma cruzi*, which causes Chagas disease in humans and dogs. Chagas disease is a major public health problem throughout Central and South America, and is considered a 'Neglected Tropical Disease' due to lack of adequate research funding and preventive measures. Raised awareness suggests the bugs and parasite are more common in the southern United States than previously thought, and our research will help to understand the ecology of disease emergence.

Many conditions can determine where kissing bugs may come into contact with humans and dogs and whether or not they transmit this deadly parasite. Even if a bug is not carrying the parasite, a bite can cause an allergic reaction.

Chagas Disease Transmission

The parasite *Trypanosoma cruzi* can live in the digestive system of the kissing bug. After taking a bloodmeal from an animal or human, the bug defecates, and the parasite is deposited in the feces. Open wounds (such as the bite that the bug just created), mucous membranes, or ingestion are all ways that the parasite can enter the body and cause infection.

Chagas disease initially causes a localized reaction (see photo above right), but the parasite can later affect the heart and digestive tract, and can ultimately cause death. No vaccines exist, and medications are few and not always effective.

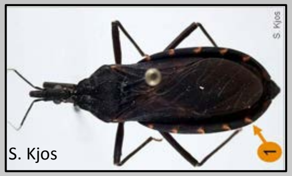

Triatoma indictiva
Adult female

Allergic Reactions

The bite of a kissing bug can cause a moderate-to-severe allergic reaction. Kissing bugs prefer to bite near the mouth and eye regions that are exposed while we sleep and can cause a localized swelling and allergic response similar to other insect bites. The reaction can be painful, and some individuals may have a heightened response that can result in anaphylactic shock and the need for hospitalization.

It is also very important not to touch kissing bugs with bare hands, and to thoroughly clean all surfaces that may have come into contact with kissing bugs and their feces.
