# Supplementary material for: Combining Public Health Education and Disease Ecology Research: Using Citizen Science to Assess Chagas Disease Entomological Risk in Texas
Source: PLoS Negl Trop Dis. 2015 Dec 10;9(12):e0004235. doi: 10.1371/journal.pntd.0004235 (PMC4687635; doi:10.1371/journal.pntd.0004235)
Supplement: S2 File — Tri-fold pamphlet about canine Chagas disease used in support of citizen science initiative in Texas. (PDF) [file pntd.0004235.s002.pdf]

Prevention

Prevent dogs from eating bugs
House dogs indoors at night
Prevent dogs from eating potentially infected animals (mice, rats, etc.)
Test breeding females, to prevent congenital transmission

Client education

Although direct transmission from dogs to humans has not been reported, infection in dogs indicates the local presence of infected vectors, which may present an increased risk of vector-borne transmission to humans.

If you or a client see a kissing bug and you would like confirmation of the species and to submit it for testing for our research purposes, our lab will accept samples.

Use a glove or small plastic bag to catch the bug to avoid direct contact. Store the bug in a sealed plastic bag, vial, or other container. All surfaces with which the bug came into contact should be thoroughly cleaned with a bleach solution. You may contact us with questions or for submissions.

It is very important for us to know exactly where the bug was found, what the bug was doing (flying, walking, etc.), the date, and the time.

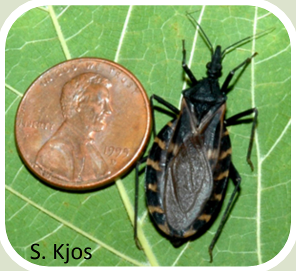

Triatoma gerstaeckeri
Kissing bug adult female

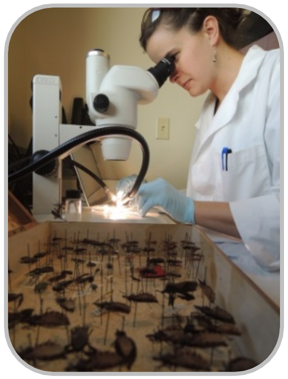

Chagas disease research at Texas A&M University

Researchers in the College of Veterinary Medicine and Department of Entomology have new projects underway to understand the ecology of kissing bugs, *Trypanosoma cruzi*, and risk of infection in humans and dogs. Samples from bugs and potentially infected animals are tested for the parasite, and the results are being used to answer questions related to whether Chagas disease will become a disease of major public health concern in the United States.

Research Team at Texas A&M

KissingBug@cvm.tamu.edu
(979) 458-4924

Rachel Curtis, NSF Graduate Research Fellow
Carolyn Hodo, DVM, Anatomic Pathology Resident
Sarah Hamer, PhD, DVM, Assistant Professor
Karen Snowden, DVM, DACVM, Professor
Gabriel Hamer, PhD, Clinical Assistant Professor

VETERINARY MEDICINE & BIOMEDICAL SCIENCES
TEXAS A&M UNIVERSITY

AGRICULTURE & LIFE SCIENCES
TEXAS A&M UNIVERSITY

Chagas disease in Texas

Information for veterinary professionals with canine patients

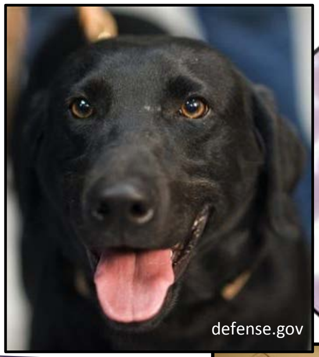

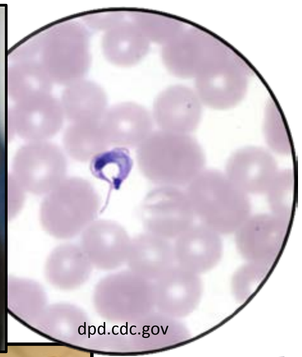

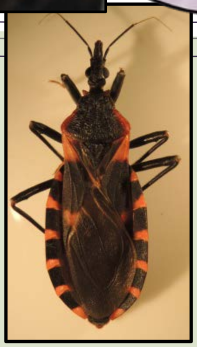

VETERINARY MEDICINE & BIOMEDICAL SCIENCES
TEXAS A&M UNIVERSITY

"Discovering tomorrow's health solutions today."

Chagas disease: the facts

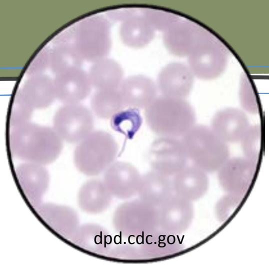

Trypanosoma cruzi, causative agent of Chagas disease

The vector-borne protozoan parasite *Trypanosoma cruzi* causes Chagas disease in humans and dogs. The nocturnal insect vector, commonly known as the kissing bug, can transmit the parasite to hosts by biting and subsequently defecating near the site of the bite. The parasites contained in the feces of the bug infect the dog via mucous membranes or breaks in the skin. Dogs can also acquire infection by eating infected bugs. Congenital transmission is also possible.

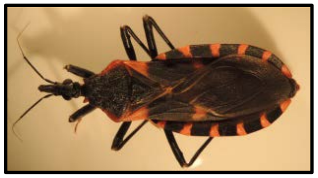

***Triatoma sanguisuga*,**
A kissing bug vector of Chagas disease

At-risk populations

Sport-type and working dogs
Young dogs
Puppies from diseased mothers
Kennel environments
Dogs that sleep outdoors

Clinical Signs

Sudden death can occur at any stage of the disease.

Acute Phase: fever, anorexia, lethargy, lymphadenopathy, hepatomegaly, splenomegaly, cardiac conduction abnormalities or arrhythmias

Latent Phase: no clinical signs, but sudden death may occur

Chronic Phase: congestive heart failure, dilated cardiomyopathy (DCM) and arrhythmias

In congenitally affected puppies, the symptoms may be slightly different.

Differential Diagnoses

Leishmania
Other causes of DCM

Diagnosis

Diagnosis is based on a high index of suspicion, combined with compatible clinical signs and echocardiographic and ECG findings.
The standard method of diagnosis is through serology (IFA). Identification of organisms on blood smear is possible during the acute phase, but not always reliable. In-house rapid serologic tests are not yet approved for use in the United States.

The Texas A&M Veterinary Medical Diagnostic Laboratory (TVMDL) offers testing for Chagas disease. A 1cc serum sample is acceptable for serology testing to determine antibody titers. More information can be found at www.tvmdl.tamu.edu

Treatment

There are no effective drugs to cure Chagas disease. Therapy directed against the organism does not alter the course of the disease in the chronic stage. Focus is on symptomatic treatment of heart failure and arrhythmias.
Drug-development studies are on-going.

Chagas disease recently became a *notifiable condition* in Texas.
Cases should be reported to your regional health department within 1 week.
www.dshs.state.tx.us/idcu/health/zoonosis/forms/animal
